# Extended ECG Monitoring in Patients with Hypertrophic Cardiomyopathy: The Tempo-HCM Study

**DOI:** 10.3390/jcm14207432

**Published:** 2025-10-21

**Authors:** Juan Caro-Codón, Sergio Castrejón, Rosalía Cadenas, Carlos Casanova, Andrea Vélez, Mayte Basurte, Gemma Lacuey, Vicente Climent, Óscar Salvador, Andrea Severo-Sánchez, Luis Fernández, Esther Pérez-David, Rafael Peinado, Silvia Valbuena-López, Gabriela Guzmán, Álvaro Jiménez-Mas, Raúl Moreno, Jose L. Merino

**Affiliations:** 1 Cardiology Department, University Hospital La Paz, IdiPaz, Paseo de la Castellana 261, 28046 Madrid, Spainsilviacayetana.valbuena@salud.madrid.org (S.V.-L.);; 2 Cardiology Department, University Hospital Infanta Sofía, San Sebastián de los Reyes, 38702 Madrid, Spain; 3 Cardiology Department, Hospital Universitario de Navarra, 31008 Pamplona, Spain; 4 Cardiology Department, Hospital General de Alicante, 03010 Alicante, Spain; 5 Cardiology Department, Hospital Universitario de Torrejón, Torrejón de Ardoz, 28850 Madrid, Spain; oscarsalvador24@gmail.com (Ó.S.);; 6 Cardiology Department, Hospital Universitario 12 de Octubre, Imas12, CIBER-CV, 28041 Madrid, Spain; 7 Institute of Medical and Molecular Genetics, University Hospital La Paz, IdiPaz, Paseo de la Castellana 261, 28046 Madrid, Spain

**Keywords:** hypertrophic cardiomyopathy, sudden cardiac death, atrial fibrillation, non-sustained ventricular tachycardia, risk stratification, electrocardiographic monitoring

## Abstract

**Highlights:**

**What’s known?**

- Thromboembolic events and SCD are two well-established complications of HCM linked, respectively, to atrial and ventricular arrhythmias.
- Clinical practice guidelines recommend monitoring using periodic 24–48 h Holter to aid clinicians in decisions regarding oral anticoagulation to prevent thromboembolic events and ICD placement for SCD prevention.
- Extended electrocardiographic monitoring well beyond 24–48 h has proven valuable in different clinical scenarios but has not been studied in patients with HCM.

**What’s new?**

- Extended ECG monitoring significantly enhances the detection of relevant arrhythmias in patients with HCM.
- NSVT are detected in most low-risk HCM patients using this technique.
- Further research is needed before routinely including findings from extended monitoring in the risk stratification process for SCD, as this strategy may compromise HCM-SCD calculator specificity.
- Extended monitoring might improve AF screening in HCM.

**Abstract:**

**Background/Objectives:** Current guidelines recommend 24–48 h Holter for risk stratification and atrial fibrillation (AF) screening in hypertrophic cardiomyopathy (HCM). However, the limited duration of this approach may not provide optimal sensitivity. In addition, extended ECG monitoring has been demonstrated to be more effective in detecting arrhythmias in other clinical entities. We aimed to assess the utility of extended ECG monitoring for 30 days in a non-high-risk cohort of HCM patients. **Methods:** We conducted a prospective multicentre study with 113 non-high-risk HCM patients who underwent 30-day ECG monitoring with a dedicated device. We compared the detection of relevant arrhythmias (AF, atrial flutter, and non-sustained ventricular tachycardia) during 30-day monitoring with the findings observed during the first 24 h. **Results:** Extended ECG monitoring detected relevant arrhythmias in 63.7% of patients, compared with 12.4% during the first 24 h (*p* < 0.001). This difference was mainly driven by non-sustained ventricular tachycardia (NSVT) (61.1% vs. 8.9%, *p* < 0.001). Atrial fibrillation episodes were detected in 10.6% of patients after completing prolonged monitoring vs. 6.2% during the first 24 h (*p* = 0.066). Extended monitoring resulted in a reclassification of 21.2% of patients to a higher sudden cardiac death (SCD) risk category using the HCM-SCD calculator. **Conclusions:** Extended ECG monitoring significantly enhances the detection of arrhythmias in HCM. Using this technique, NSVT were detected in most patients of a non-high-risk HCM cohort. Further investigation is warranted to determine the role of extended monitoring in SCD risk stratification and AF screening.

## 1. Background

Contemporary studies suggest a favourable global prognosis for hypertrophic cardiomyopathy (HCM). Many patients remain asymptomatic throughout their lives, and the disease-related mortality rate may be as low as 0.5% per year [1]. However, some will develop major complications during long-term clinical follow-up. Given its status as the most common genetic cardiac disease, with an estimated prevalence between 1:200 and 1:500 [2], the burden of related adverse effects constitutes a significant public health concern. Specifically, thromboembolic events and sudden cardiac death (SCD) are two well-established complications that have been linked, respectively, to atrial and ventricular arrhythmias [3,4]. Based on this rationale, clinical practice guidelines recommend routine monitoring using periodic 24–48 h Holter at long-term intervals [5,6,7]. Therefore, detecting atrial fibrillation (AF) or non-sustained ventricular tachycardia (NSVT) remains crucial for risk stratification in HCM, guiding clinicians in decisions regarding oral anticoagulation to prevent thromboembolic events or implantable cardioverter-defibrillator (ICD) placement for primary SCD prevention.

Extended electrocardiographic monitoring well beyond 24–48 h has proven valuable in several clinical scenarios, such as post-cryptogenic stroke assessment [8,9] or following AF ablation [10]. In addition, previous research has demonstrated that extending monitoring time improves sensitivity to detect atrial arrhythmias [11,12,13]. However, no prospective and properly designed studies have been conducted to assess the utility of non-invasive prolonged ECG monitoring in patients with HCM.

The TEMPO-HCM registry is a prospective, multicentre observational study that aim to evaluate the usefulness of 30-day monitoring in non-selected non-high-risk patients diagnosed with HCM. We compared the diagnostic yield of this strategy with the findings observed during the first 24 h of monitoring.

## 2. Methods

### 2.1. Study Design

The TEMPO-HCM registry is an investigator-initiated prospective, multicentre observational study. We recruited consecutive non-high risk adult patients diagnosed with HCM who also had a clinical indication for conventional Holter ECG monitoring either for AF screening or as part of SCD risk stratification [5,6,7]. The study protocol was approved by the Ethics Committee at each centre (PI-4673), and written informed consent was obtained from all subjects before enrolment. All practices were conducted in accordance with the Strengthening the Reporting of Observational Studies in Epidemiology (STROBE) statement [14].

### 2.2. Patients

Eligible patients were aged 18 years or older and had a wall thickness of ≥15 mm in one or more left ventricular myocardial segments as measured by any imaging technique, that was not explained by loading conditions. Alternatively, the diagnosis of HCM could also be established in first-degree relatives for patients exhibiting otherwise unexplained left ventricular wall thickness of ≥13 mm in one or more myocardial segments. Overt phenocopies of HCM, such as cardiac amyloidosis or Fabry disease, were excluded. Patients who already had an ICD or a SCD risk at 5 years over 6%, as estimated with the HCM-SCD calculator [15,16], were also excluded, as we aimed to study a non-high-risk population. Patients were consecutively included during their assessment at a dedicated cardiology clinic in one of the five participating centres. After inclusion, all pseudonymized clinical information was incorporated into a specific electronic database [17].

### 2.3. ECG Monitoring

All enrolled patients underwent extended electrocardiographic monitoring using a dedicated device (Nuubo, Nuubo Wearable Medical Technologies^®^, Madrid, Spain), based on a recorder and a textile garment that adapts to the patient’s chest dimensions. The garment includes four non-metallic electrodes in contact with the skin, allowing continuous recording of a 2-lead electrocardiogram [18,19]. Patients were instructed to recharge the device battery every day during shower time (15–20 min) and wear the recording system throughout the rest of the time to maximize the detection of arrhythmias. All traces were centrally analyzed by an experienced electrophysiologist and a clinician with dedication to inherited cardiac conditions, and arrhythmic events were adjudicated using standard definitions: non-sustained ventricular tachycardia (three or more ventricular beats with a rate exceeding 120 bpm and a duration of less than 30 s) and AF (irregular tachycardia with QRS morphology similar to the baseline, absence of organized atrial activity, and a duration exceeding 30 s). Any disagreement was jointly reviewed, and a consensus decision was made.

### 2.4. Outcomes

The primary aim of the study was to compare the detection of relevant arrhythmias (a composite of AF, atrial flutter, and NSVT) during a whole 30-day extended ECG monitoring with the findings observed during the first 24 h. The secondary outcomes included the assessment of individual components of the primary outcome, and an exploratory analysis comparing the 5-year SCD risk estimated by the HCM-SCD calculator [15,16].

### 2.5. Additional Testing and Follow-Up

Although not mandatory, all participating centres were encouraged to perform additional conventional 24 h ECG Holter close to the time of inclusion. A comprehensive imaging assessment with transthoracic echocardiography and cardiac magnetic resonance was also recommended. HCM AF score, a clinical predictive model for AF validated in HCM [20], was calculated for all patients.

### 2.6. Statistical Analysis

Categorical variables are described using absolute numbers and proportions. They were analyzed using the McNemar test for paired samples. Continuous variables are described as mean ± standard deviation or median (interquartile range). Normality was assessed using the Shapiro–Wilk test and visual examination of histograms and Q-Q plots. For normally distributed continuous variables, paired *t*-tests were used for comparisons, while the Wilcoxon test was employed for non-normally distributed variables. Logistic regression was used to identify factors that predicted both the composite of relevant arrhythmias and NSVT during extended monitoring. We included as candidate variables for the multivariable regression those that presented a significant association in the univariate analysis as well as the baseline 5-year risk for SCD using HCM-SCD (including in the calculation the presence or absence of NSVT prior to the entrance in the study). The incidence of arrhythmias during electrocardiographic monitoring was described using Kaplan–Meier analysis. No imputation was performed for missing data and a pairwise deletion approach was performed as appropriate in the analysis.

The sample size calculation was based on studies focused on the prevalence of atrial and ventricular arrhythmias in patients with HCM, as well as those that have demonstrated increased sensitivity to extended monitoring in different patient populations [15,20,21,22,23]. However, there is a lack of information regarding the coexistence of both types of arrhythmias in samples of patients with HCM. Hypothesizing a prevalence of clinically significant arrhythmias (AF and NSVT) of approximately 30% during 24 h monitoring and empirically estimating that extended monitoring can identify events in 50% of patients, with a two-sided alpha risk of 5%, approximately 10% loss to follow-up, and a statistical power of 80%, the sample size was estimated to be 103 patients.

Data analysis was performed using the statistical software Stata v14.2 (StataCorp, College Station, TX, USA) and R (v4.1.1). A two-sided *p*-value of <0.05 was considered statistically significant for all analyses.

## 3. Results

### 3.1. Patients Demographics and Characteristics

From February 2021 to January 2023, a total of 130 patients from five centres across Spain were screened to enter the study. Seventeen patients were excluded (one patient died due to non-cardiovascular causes before monitoring, one patient did not meet diagnostic criteria for HCM after cardiac MRI indicated by the study investigators and fifteen patients finally refused to undergo extended monitoring or were not compliant with the study instructions). Finally, 113 patients were included in the present analysis. The main baseline characteristics are displayed in Table 1. Median age was 57.9 (48.0–67.1) years and the majority were male (77.9%). Included patients were mainly probands (75.2%), 99 (87.6%) underwent genetic testing, and 36 (36.4% of those tested) were carriers of a pathogenic or likely pathogenic genetic variant, mainly related to sarcomeric genes. Most patients were in NYHA functional class I and the majority (79, 69.1%) had no known classical risk factors [7] associated with SCD. Median 5-year risk of SCD prior to the entrance in the study was 1.89 (1.36–2.60)%, with 104 patients (92.0%) considered to be at low risk and 9 (8.0%) at intermediate risk. In addition, only 16 (14.2%) had documented NSVT prior to the entry in the study and 18 (15.9%) had AF (mainly paroxysmal AF).

### 3.2. Monitoring Results

**Figure 1:**
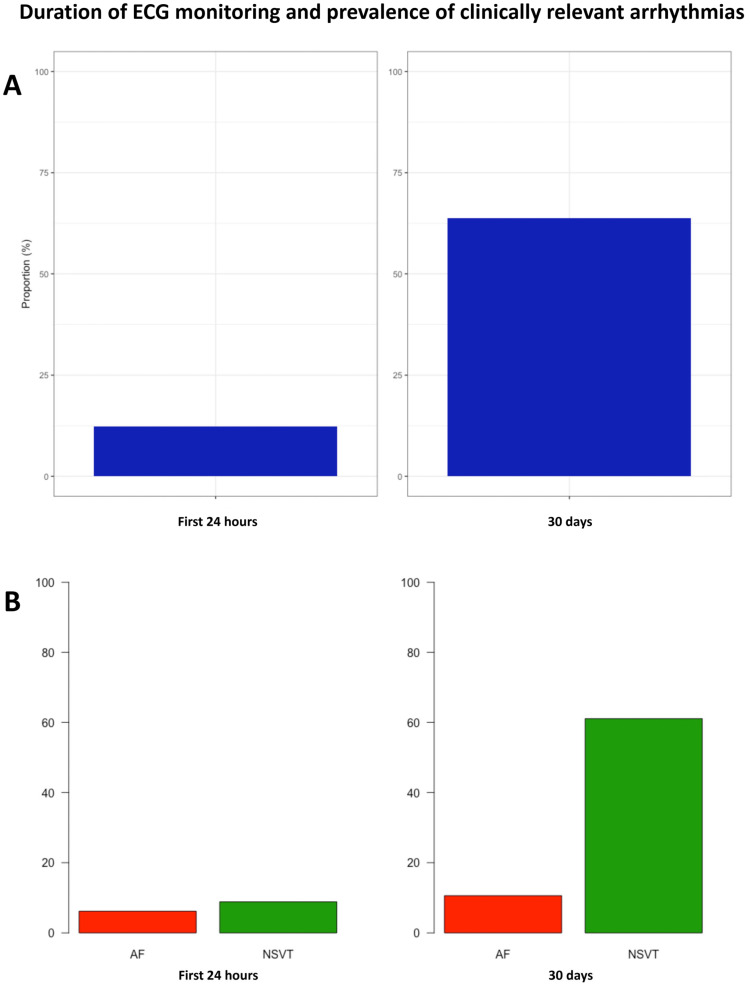
Panel (**A**) shows the prevalence of a composite outcome of NSVT and AF after 24 h and 30 days of monitoring. Panel (**B**) shows the prevalence of NSVT and AF separately.

**Figure 2:**
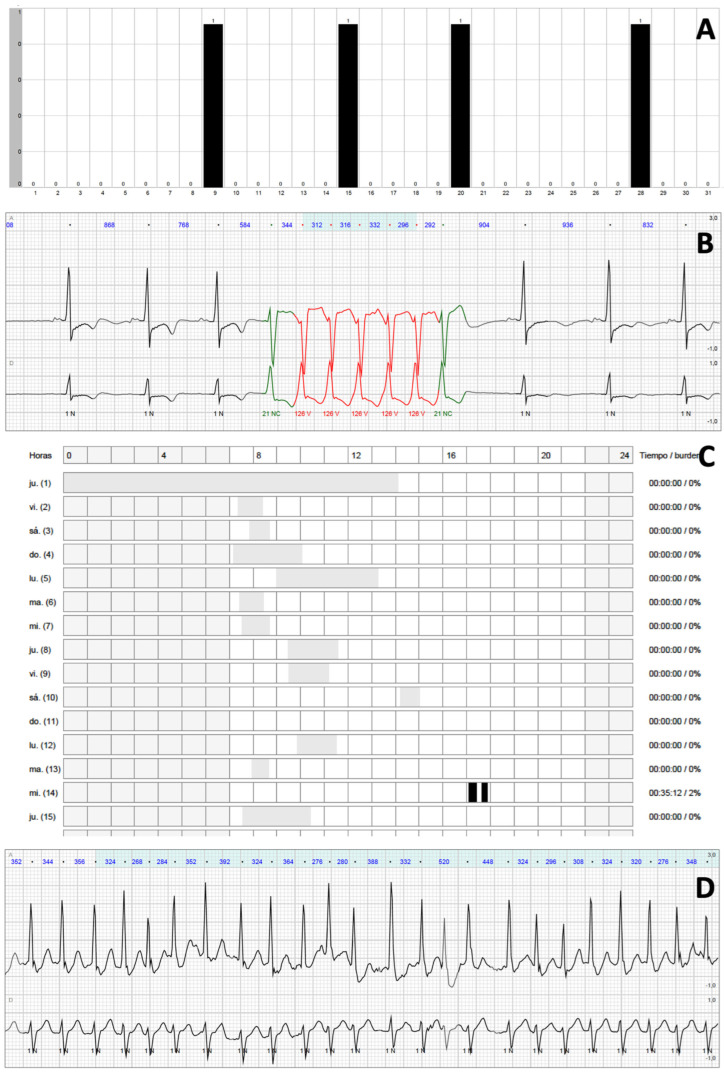
Illustrative findings in two patients after extended monitoring. Panels (**A**,**B**) were obtained from a patient with four episodes of fast NSVT well beyond 24–48 h of monitoring (at days 9, 15, 20 and 28). Panels (**C**,**D**) illustrate another patient with a new diagnosis of paroxysmal AF in day 14.

Each patient underwent extended ECG monitoring during a median of 684 (647–721) h. Excluding those periods in which the quality of the signal was not optimal, a median of 586 (482–652) h per patient or 19.5 (16.1–21.7) h per day and patient were available for the analysis. Results from the primary outcome are shown in Figure 1. Overall, extended ECG monitorization with a dedicated device for 30 days detected relevant arrhythmias in 72 patients (63.7%) vs. 14 patients (12.4%) during the first 24 h of monitoring (*p* < 0.001). This significant difference was mainly driven by NSVT [69 (61.1%) vs. 10 (8.9%), *p* < 0.001]. AF episodes were detected in 12 patients (10.6%) after completing prolonged monitoring vs. 7 patients (6.2%) during the first 24 h (*p* = 0.066). No episodes of atrial flutter were observed. The results were homogenous throughout all of the study centres (Supplementary Table S1). Illustrative reports of patients with relevant findings are shown in Figure 2. Additional conventional Holter monitoring was performed in 96 patients (85.0%), 17 of whom (17.7%) presented NSVT (*p* = 0.044 when compared with NSVT detection during the first 24 h of extended monitoring), and 5 presented AF (all 5 patients with prior permanent AF).

Patients with relevant arrhythmias during extended monitorization were older [60.6 (51.1–71.3) vs. 53.3 (41.6–61.0) years, *p* = 0.004], had more frequent history of prior NSVT [15 (20.8%) episodes vs. 1 (2.4%), *p* = 0.009], larger left atrial diameters (42.9 ± 7.5 vs. 38.9 ± 6.9 mm, *p* = 0.007), thicker left ventricular walls [18 (16–20) vs. 17 (14.5–19) mm, *p* = 0.039], and more frequent detection of late gadolinium enhancement in cardiac MRI [46 (85.2%) vs. 12 (37.5%), <0.001] (Table 2). After multivariable logistic regression analysis, the latter was the only independent predictor for the detection of arrythmias during extended monitoring (Supplementary Table S2).

### 3.3. NSVT

A total of 418 episodes of NSVT were detected in 69 patients (61.1%), with a median of 3 (1–6) episodes per patient, a median heart rate of 153 (135–172) bpm and a median duration of 8 (4–12) beats. Details regarding the differences between patients who experienced NSVT and those who did not can be found in Supplementary Table S3. Patients with NSVT were older [60.6 (50.8–70.8) vs. 54.4 (41.9–61.6), *p* = 0.010], had more frequent history of prior NSVT [15 (21.7%) vs. 1 (2.3%), *p* = 0.004], larger LA diameters (42.8 ± 7.6 vs. 39.3 ± 7.1, *p* = 0.017), and more frequent detection of late gadolinium enhancement in cardiac MRI [43 (84.3%) vs. 15 (42.9%), *p* < 0.001]. As with all relevant arrhythmias, late gadolinium enhancement was the only independent predictor of NSVT after multivariable logistic regression (Supplementary Table S4).

**Figure 3:**
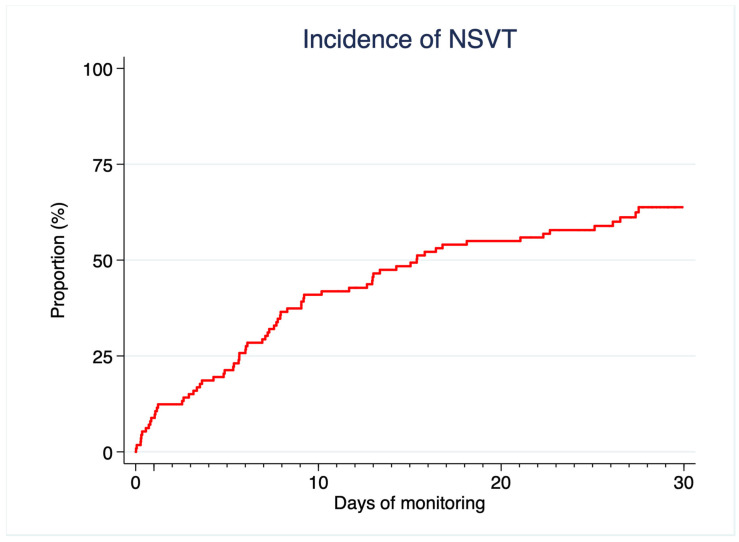
Kaplan–Meier survival analysis for the incidence of NSVT during extended monitoring.

Incidence of NSVT as described by the Kaplan–Meier failure function is depicted in Figure 3. Interestingly, occurrence of a first episode of NSVT continued throughout the study period and did not actually reach a clear plateau upon completion of 30-day monitoring. The global profile of NSVT in patients with episodes detected prior to 24 h was somehow more aggressive, with more frequent, faster, and prolonged bursts of ventricular arrhythmia (Table 3). These patients presented a higher 5-year baseline risk for SCD assessed by HCM-SCD, mainly driven by a more frequent prior history of NSVT, which was the other significant predictor for early ventricular arrhythmias during extended monitoring (Supplementary Table S5).

**Figure 4:**
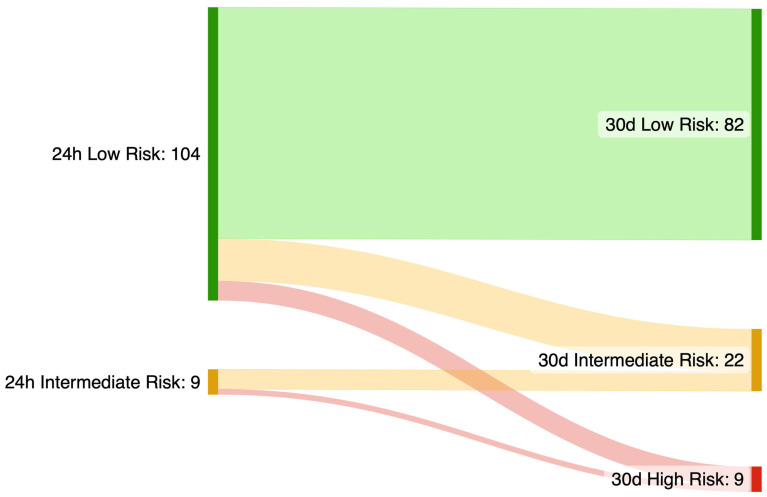
Sensitivity analysis of the HCM-SCD calculator incorporating findings from 30-day monitoring.

We additionally performed an exploratory analysis estimating the 5-year SCD risk with the HCM-SCD calculator (Figure 4). Median estimated SCD risk at 5 years was 1.76 (1.32–2.48)% using the first 24 h data, versus 2.77 (1.97–4.05)% using extended monitoring (*p* < 0.001). Extended monitoring resulted in a reclassification of 24 patients (21.2%) to a higher SCD risk category, with 15 (13.3%) additional cases in which an ICD may be considered and 9 (8.0%) additional cases in which an ICD should be considered.

### 3.4. Atrial Fibrillation

**Figure 5:**
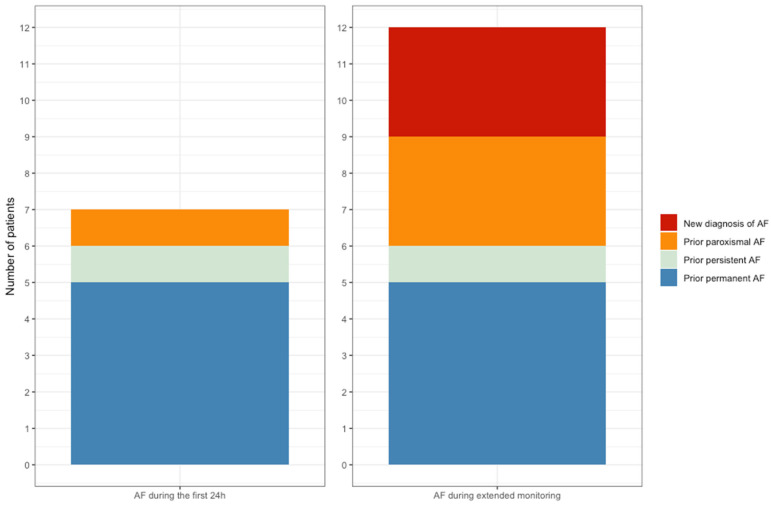
Prevalence and characteristics of AF episodes detected after 24 h and 30 days monitoring.

During the first 24 h, AF was detected in five patients who had a previous diagnosis of permanent AF, as well as in two additional subjects with paroxysmal and persistent AF who were in sinus rhythm at the start of the monitoring period. Extended monitoring led to a new diagnosis of AF in three patients without prior history of this arrhythmia [2.7% of the study sample, median burden 28.6 (0.6–84.5) h], as well as two more subjects with previous paroxysmal AF (Figure 5). The three new diagnoses of AF were made among patients with intermediate or high risk for developing AF as assessed by the HCM AF risk scale (Supplementary Figure S1). The difference between the proportion of patients with episodes of AF detected during 24 h and 30 days did not achieve statistical significance (*p* = 0.063).

## 4. Discussion

Our data provides the first evidence that 30-day extended electrocardiographic monitoring in a non-high-risk cohort of patients with HCM significantly enhances the detection of relevant arrhythmias as compared with a more conventional 24 h approach. Over 60% of a predominantly low-risk sample of patients had relevant arrhythmias during the study procedures. This result was due to a very high prevalence (61.6%) of NSVT, which was higher than expected for this population. The identification of NSVT during extended monitoring resulted in a significantly higher 5-year risk of SCD as estimated by the HCM-SCD calculator, potentially reclassifying 21.2% of the patients to a higher risk category. In addition, extended monitoring resulted in numerically more diagnoses of AF.

NSVT in HCM has been historically associated with SCD [21,24,25,26,27]. Even though age has been acknowledged as a relevant risk modifier, detection of NSVT has been given relevance in the whole population of HCM patients and has been incorporated in the risk stratification process via the HCM-SCD calculator endorsed by the ESC guidelines [5]. Prevalence of NSVT in 24–48 h Holter monitoring, including those from the derivation and validation cohorts of the HCM-SCD calculator, has been described in the range of 17–32% [4,15,16]. However, data from single-centre retrospective studies including patients with a high-risk profile or already carrying an ICD suggest that this prevalence may be higher in certain subgroups [28,29].

On the other hand, patients with HCM are at a higher risk for stroke and systemic embolism [3]. It has been hypothesized than severe left atrial remodelling in HCM may constitute a substrate for thromboembolism even in the absence of AF [30], but data from large observational studies demonstrate the relevance of detecting AF in the prediction of thromboembolic events [3,22,31]. Real life experience demonstrates that there is an interaction between both conditions that, when they coexist, acts through synergistic effects and results in a higher risk of developing left atrial thrombus [32].

Our findings suggest that the true prevalence of NSVT in patients with HCM, even in those theoretically considered to be at low risk of SCD, is higher than previously thought. This raises questions about the underlying pathophysiology and prognostic power of these arrhythmias: a phenomenon which is present in the majority of patients with a certain condition is unlikely to adequately predict a rare complication of the disease. The lack of a plateau in the incidence of NSVT and the fact that the median time for valid ECG analysis in each patient was 19.6 (16.1–21.7) h per day, imply that the real prevalence of these arrhythmias might have been even higher if we had achieved uninterrupted 30-day—or even more prolonged—monitoring. The relevant risk prediction value might not be the binary finding of whether a patient has NSVT or not, but rather its specific characteristics (i.e., the global burden of NSVT or its maximum heart rate) as well as its interactions with other phenotypic traits [33,34]. This was suggested by prior research in high-risk patients already carrying an ICD, in which faster, longer, and more repetitive runs of NSVT were predictive of ICD-treated arrhythmias [28]. Thus, extended monitoring may be useful for personalizing arrhythmic risk stratification through a better characterization of individual episodes in each patient. However, it is important to consider that HCM-SCD was developed and validated in cohorts assessed with conventional 24–48 h Holter and a theoretically much lower prevalence of NSVT [15,16]. We think that caution is needed in the evaluation of findings derived from prolonged periods of monitoring, because overinterpretation may lead to an inaccurate risk stratification. Contemporary use of wearable devices with the capability to track vital signs and detect heart rate and heart rhythm during long periods of time may pose an increasing challenge to clinicians who attend patients with HCM. Further research is needed to clarify to what extent information obtained via these devices compromises the HCM-SCD calculator specificity [35,36].

On the other hand, we believe that there is a trend in this study towards a potential benefit in the use of extended monitoring for the screening of AF in HCM. Although our sample is small and does not have the statistical power to specifically assess this aspect, our findings are consistent with those observed in other clinical settings. The identification of previously undiagnosed AF in patients with HCM has a direct and immediate impact on management, as it opens the opportunity for oral anticoagulation to prevent thromboembolic events. Indeed, data from observational and randomized studies have demonstrated that AF detection in patients with risk factors for thromboembolism improves with the duration of ECG monitoring, also considering strategies with wearable devices [8,9,37]. Even though there has been debate regarding the optimal management of asymptomatic device-detected AF [38], recent studies in non-specific populations of patients with subclinical AF have shown that treatment with direct oral anticoagulants results in a lower risk of stroke or systemic embolism [39,40]. Given that HCM constitutes a high-risk substrate, failure to identify this arrhythmia may result in a lack of opportunity to start oral anticoagulation and this may jeopardize long-term prognosis. Rather than a definitive finding, our results should be interpreted as a signal that warrants further investigation, underscoring the potential role of extended ECG monitoring in enhancing AF detection and optimizing preventive strategies in this high-risk population. We believe that the use of tools like HCM-AF score may aid in the selection of patients with higher pre-test probability of AF in order to develop a cost-effective screening program [20].

Specifically addressing resource implications and implementation barriers, extended ECG monitoring over 30 days entails greater upfront device and analysis costs than conventional Holter monitoring and generates larger data volumes that require adjudication and secure storage. However, economic analyses in related clinical contexts indicate that prolonged external monitoring can be cost-neutral or cost-saving in some systems, owing to higher diagnostic yield, fewer repeat studies, and streamlined workflows; national guidance in the UK has similarly concluded that longer-duration patch monitoring may be cost-saving or cost-equivalent compared with 24 h Holter, while calling for additional real-world evidence. At the same time, cost-effectiveness depends on downstream management: in HCM, the detection of AF has immediate therapeutic consequences because oral anticoagulation is recommended when AF is documented, potentially averting thromboembolic events. Notwithstanding, HCM-specific economic evaluations of extended non-invasive monitoring are not yet available and should be addressed in future studies; practical barriers—patient adherence, reimbursement, and the need for centralized analysis—must also be considered when planning implementation.

This study has several limitations. First, the sample size was relatively small. However, we defined broad inclusion criteria and obtained the participation of different centres in order to improve its external validity. Second, the monitoring was intermittent and dependent on patient compliance, which could have decreased sensitivity for the detection of arrhythmias. This would likely result in underestimation rather than overestimation of event rates. Third, the study was restricted to a predominantly low-risk population, providing no information on patients with a higher risk profile. Fourth, NSVT definition was selected to be consistent with prior HCM literature [6,9,15,16]. However, we acknowledge that it differs from the definition stated in the 2022 ESC guidelines for the management of patients with ventricular arrhythmias [41], in which a 120 bpm limit was not included. Fifth, long-term follow-up data are not yet available, but a long-term follow-up of these patients is planned.

## 5. Conclusions

In conclusion, extended ECG monitoring for 30 days significantly improved the detection of arrhythmias in patients with HCM. Using this technique, NSVT were detected in a majority of patients within a non-selected, non-high risk HCM cohort. However, the clinical interpretation of these NSVT episodes—particularly their incorporation into established SCD risk models—requires caution and further validation in larger, outcome-based studies. Additionally, the role of extended monitoring in refining AF screening for HCM warrants further exploration, as it may represent an actionable benefit of extended monitoring in this population.

## Figures and Tables

**Table 1:** Baseline characteristics.

Variable	All Patients (n = 113)
Male sex (%)	88 (77.9)
Age (years)	57.9 (48.0–67.1)
Hypertension (%)	54 (47.8)
Diabetes (%)	13 (11.5)
Dyslipidemia (%)	57 (50.4)
Cerebrovascular disease (%)	6 (5.3)
PAD (%)	2 (1.8)
Chronic kidney disease (%)	3 (2.7)
COPD (%)	5 (4.4)
Coronary artery disease (%)	6 (5.3)
Valvular heart disease (%)	12 (10.6)
ACEi/ARB (%)	42 (37.2)
Beta blockers (%)	65 (57.5)
Dysopiramide (%)	5 (4.4)
Other antiarrhythmics (%)	3 (2.7)
Antiplatelets (%)	16 (14.2)
Anticoagulation (%)	21 (18.6)
Age at diagnosis	51.2 (40.5–60.6)
Family history of SCD (%)	16 (14.2)
P/LP variant carrier (%)	36 (36.4)
Proband status (%)	85 (75.2)
NYHA class (%)	
I	77 (68.1)
II	33 (29.2)
III	3 (2.7)
Prior HF decompensations (%)	10 (8.9)
Prior syncope (%)	6 (5.3)
Palpitations (%)	31 (27.4)
Prior atrial fibrillation (%)	18 (15.9)
Type of AF (%)	
Paroxysmal	10 (55.5)
Persistent	3 (16.7)
Permanent	5 (27.8)
Prior AF ablation (%)	7 (38.9)
History of NSVT (%)	16 (14.2)
Baseline 5-year risk SCD (%)	1.89 (1.36–2.60)
ASA (%)	4 (3.5)
Septal myectomy (%)	3 (2.7)

Abbreviatures: PAD Peripheral artery disease; COPD Chronic obstructive pulmonary disease; ACEi Angiotensin converting enzyme inhibitors; ARB Angiotensin II receptor blockers; P/LP Pathogenic/Likely Pathogenic; HF Heart failure; AF Atrial fibrillation; NSVT Non-sustained ventricular tachycardia; ASA Alcohol septal ablation. Notes: A total of 99 patients (87.6%) underwent genetic testing. Valvular heart disease refers to moderate or severe disease at the mitral or artic position.

**Table 2:** Differences between patients with and without clinically relevant arrhythmias.

Variable	All Patients (n = 113)	CR Arrhythmia (n = 72)	No CR Arrhythmia (n = 41)	*p* Value
Male sex (%)	88 (77.9)	59 (81.8)	29 (70.7)	0.167
Age (years)	57.9 (48.0–67.1)	60.6 (51.1–71.3)	53.3 (41.6–61.0)	0.004
Hypertension (%)	54 (47.8)	34 (47.2)	20 (48.8)	0.873
Diabetes (%)	13 (11.5)	9 (12.5)	4 (9.8)	0.766
Dyslipidemia (%)	57 (50.4)	33 (45.8)	24 (58.5)	0.194
Cerebrovascular disease (%)	6 (5.3)	5 (6.9)	1 (2.4)	0.414
PAD (%)	2 (1.8)	1 (1.4)	1 (2.4)	1.000
Chronic kidney disease (%)	3 (2.7)	2 (2.8)	1 (2.4)	1.000
COPD (%)	5 (4.4)	3 (4.2)	2 (4.9)	1.000
Coronary artery disease (%)	6 (5.3)	5 (6.9)	1 (2.4)	0.414
Valvular heart disease (%)	12 (10.6)	9 (12.5)	3 (7.3)	0.531
ACEi/ARB (%)	42 (37.2)	29 (40.3)	13 (31.7)	0.365
Beta blockers (%)	65 (57.5)	44 (61.1)	21 (51.2)	0.306
Dysopiramide (%)	5 (4.4)	4 (5.6)	1 (2.4)	0.651
Other antiarrhythmics (%)	3 (2.7)	3 (4.7)	0 (0.0)	0.552
Antiplatelets (%)	16 (14.2)	9 (12.5)	7 (17.1)	0.503
Anticoagulation (%)	21 (18.6)	15 (20.8)	6 (14.6)	0.415
Age at diagnosis	51.2 (40.5–60.6)			
Family history of SCD (%)	16 (14.2)	10 (13.9)	6 (14.6)	0.913
P/LP variant carrier (%)	36 (36.4)	26 (39.4)	10 (30.3)	0.375
Proband status (%)	85 (75.2)	56 (77.8)	29 (70.7)	0.404
NYHA class (%)				
I	77 (68.1)	50 (69.4)	27 (65.9)	
II	33 (29.2)	20 (27.8)	13 (31.7)	0.853
III	3 (2.7)	2 (2.8)	1 (2.4)	
Prior HF decompensations (%)	10 (8.9)	7 (9.7)	3 (7.3)	0.745
Prior syncope (%)	6 (5.3)	4 (5.6)	2 (4.9)	1.000
Palpitations (%)	31 (27.4)	20 (27.8)	11 (26.8)	0.913
Prior atrial fibrillation (%)	18 (15.9)	14 (19.4)	4 (9.8)	0.284
Type of AF (%)				
Paroxysmal	10 (55.5)	7 (50.0)	3 (75.0)	
Persistent	3 (16.7)	2 (14.3)	1 (25.0)	0.436
Permanent	5 (27.8)	5 (35.7)	0 (0.0)	
Prior AF ablation (%)	7 (38.9)	6 (42.9)	1 (25.0)	1.000
History of NSVT (%)	16 (14.2)	15 (20.8)	1 (2.4)	0.009
Baseline 5-year risk SCD (%)	1.89 (1.36–2.60)	1.90 (1.38–2.91)	1.82 (1.32–2.42)	0.335
ASA (%)	4 (3.5)	1 (1.4)	3 (7.3)	0.135
Septal myectomy (%)	3 (2.7)	2 (2.8)	1 (2.4)	1.000
Imaging studies				
PLAX LA diameter (mm)	41.5 ± 7.5	42.9 ± 7.5	38.9 ± 6.9	0.007
Maximum LV thickness (mm)	17 (15–20)	18 (16–20)	17 (14.5–19)	0.039
LVEF (%)	66.3 ± 7.0	65.6 ± 7.6	67.5 ± 5.6	0.180
Significant LVOT obstruction (%)	22 (19.5)	13 (18.1)	9 (22.0)	0.615
Apical aneurysm (%)	5 (4.4)	4 (5.6)	1 (2.4)	0.652
Late gadolinium enhancement (%)	58 (67.4)	46 (85.2)	12 (37.5)	<0.001
24 h Holter monitoring (n = 96)				
NSVT (%)	17 (17.7)	16 (24.6)	1 (3.2)	0.010
AF (%)	5 (5.2)	5 (7.7)	0 (0.0)	0.171
Premature atrial complexes (n)	47 (13–220)	87 (15–225)	37 (8–82)	0.255
Premature ventricular complexes (n)	32 (3–181)	36 (7–178)	16 (2–196)	0.344

Abbreviatures: PAD Peripheral artery disease; COPD Chronic obstructive pulmonary disease; ACEi Angiotensin converting enzyme inhibitors; ARB Angiotensin II receptor blockers; P/LP Pathogenic/Likely Pathogenic; HF Heart failure; AF Atrial fibrillation; NSVT Non-sustained ventricular tachycardia; ASA Alcohol septal ablation; LA Left atrium; LV Left ventricle; LVEF Left ventricular ejection fraction; MR mitral regurgitation. Notes: A total of 99 patients (87.6%) underwent genetic testing. Valvular heart disease refers to moderate or severe disease at the mitral or aortic position. Significant LVOT obstruction was defined as ≥30 mmHg as per clinical guidelines. A total of 86 patients (76.1%) underwent cardiac magnetic resonance.

**Table 3:** Profile of NSVT in patients with and without episodes during the first 24 h.

NSVT Characteristics	All Patients with NSVT (n = 69)	NSVT 0–24 h (n = 10)	NSVT 24 h-30 d (n = 59)	*p* Value
Number of NSVT per patient	3 (1–6)	10 (9–19)	2 (1–4)	<0.001
Heart rate (bpm)	153 (135–172)	170 (160–175)	148 (132–169)	0.034
Duration (beats)	8 (4–12)	12 (8–16)	8 (4–11)	0.026

Abbreviatures: NSVT Non-sustained ventricular tachycardia, bpm beats per minute.

## Data Availability

The original contributions presented in this study are included in the article/Supplementary Material. Further inquiries can be directed to the corresponding author.

## Supplementary Materials

The following supporting information can be downloaded at: https://www.mdpi.com/article/10.3390/jcm14207432/s1, Table S1: Primary and secondary outcomes assessed by each participating center; Table S2: Logistic regression analysis of univariable and multivariable predictors of clinically relevant arrhythmias; Table S3: Differences between patients with and without NSVT; Table S4: Logistic regression analysis of univariable and multivariable predictors of NSVT; Table S5: Differences between patients with early and non-early NSVT (first 24 h vs. 24 h-30 days). Figure S1: Distribution of HCM-AF score among the study population.

## Abbreviations

AF	Atrial fibrillation
HCM	Hypertrophic cardiomyopathy
ICD	Implantable cardioverter defibrillator
NSVT	Non-sustained ventricular tachycardia
SCD	Sudden cardiac death
