# Supplementary material for: Extended ECG Monitoring in Patients with Hypertrophic Cardiomyopathy: The Tempo-HCM Study
Source: J Clin Med. 2025 Oct 21;14(20):7432. doi: 10.3390/jcm14207432 (PMC12565036; doi:10.3390/jcm14207432)

Supplementary Table S1. Primary and secondary outcomes assessed by each participating center

	Centre 1 (n= 81)	Centre 2 (n=5)	Centre 3 (n=12)	Centre 4 (n=8)	Centre 5 (n=7)	p value
NSVT	50 (61.7)	4 (80.0)	7 (58.3)	5 (62.5)	3 (42.9)	0.809
AF	10 (12.4)	0 (0.0)	2 (16.7)	0 (0.0)	0 (0.0)	0.813
Total	53 (65.4)	4 (80.0)	7 (58.3)	5 (62.5)	3 (42.9)	0.713

Abbreviatures: NSVT Non-sustained ventricular tachycardia, AF Atrial Fibrillation

Supplementary Table S2 Logistic regression analysis of univariable and multivariable predictors of clinically relevant arrhythmias

	Univariable			Multivariable		
	OR (95% CI)	SE	p value	OR (95% CI)	SE	p value
Age (per 10 years increment)	1.54 (1.16-2.04)	0.22	0.003	1.31 (0.91-1.88)	0.24	0.142
History of NSVT	10.52 (1.34-82.94)	11.09	0.025	9.98 (0.96-104.20)	11.94	0.055
PLAX LA diameter (per mm)	1.08 (1.02-1.15)	0.03	0.010	1.06 (0.98-1.15)	0.42	0.120
Maximum LV thickness (per mm)	1.12 (1.00-1.25)	0.06	0.046	1.09 (0.93-1.27)	0.09	0.311
LGE in MRI	4.28 (1.87-9.78)	1.20	0.001	3.20 (1.27-8.04)	1.50	0.013
Baseline HCM SCD (per 1%)	1.26 (0.87-1.84)	0.24	0.220	0.81 (0.41-1.60)	0.28	0.543

Abbreviatures: NSVT Non-sustained ventricular tachycardia, PLAX LA Parasternal long axis left atrial, LV Left ventricle, LGE Late gadolinium enhancement, MRI Magnetic resonance imaging.

Supplementary Table S3. Differences between patients with and without NSVT

Variable	All patients (n=113)	NSVT (n=69)	No NSVT (n=44)	p value
Male sex (%)	88 (77.9)	56 (81.2)	32 (72.7)	0.292

Age (years)	57.9 (48.0-67.1)	60.6 (50.8-70.8)	54.4 (41.9-61.6)	0.010
Hypertension (%)	54 (47.8)	34 (49.3)	20 (45.5)	0.692
Diabetes (%)	13 (11.5)	8 (11.6)	5 (11.4)	1.000
Dyslipidemia (%)	57 (50.4)	30 (43.5)	27 (61.4)	0.064
Cerebrovascular disease (%)	6 (5.3)	5 (7.3)	1 (2.3)	0.402
PAD (%)	2 (1.8)	1 (1.5)	1 (2.3)	1.000
Chronic kidney disease (%)	3 (2.7)	2 (2.9)	1 (2.3)	1.000
COPD (%)	5 (4.4)	3 (4.4)	2 (4.6)	1.000
Coronary artery disease (%)	6 (5.3)	5 (7.3)	1 (2.3)	0.402
Valvular heart disease (%)	12 (10.6)	9 (13.0)	3 (6.8)	0.362
ACEi/ARB (%)	43 (38.1)	29 (42.0)	14 (31.8)	0.276
Beta blockers (%)	65 (57.5)	41 (59.4)	24 (54.6)	0.609
Dysopiramide (%)	5 (4.4)	4 (5.8)	1 (2.3)	0.647
Other antiarrhythmics (%)	3 (2.7)	2 (2.9)	1 (2.3)	1.000
Antiplatelets (%)	16 (14.2)	9 (13.0)	7 (15.9)	0.670
Anticoagulation (%)	21 (18.6)	12 (17.4)	9 (20.5)	0.683
Family history of SCD (%)	16 (14.2)	10 (14.5)	6 (13.6)	0.899
P/LP variant carrier (%)				
Proband status (%)	85 (75.2)	53 (76.8)	32 (72.7)	0.624
NYHA class (%)<br>I<br>II<br>III	 77 (68.1)<br>33 (29.2)<br>3 (2.7)	 48 (69.6)<br>19 (27.5)<br>2 (2.9)	 29 (65.9)<br>14 (31.8)<br>1 (2.3)	 0.856
Prior HF decompensations (%)	10 (8.9)	6 (8.7)	4 (9.1)	1.000
Prior syncope (%)	6 (5.3)	4 (5.8)	2 (4.6)	1.000
Palpitations (%)	31 (27.4)	18 (26.1)	13 (29.6)	0.688
Prior atrial fibrillation (%)	18 (15.9)	11 (15.9)	7 (15.9)	0.996
Type of AF (%)<br>Paroxysmal<br>Persistent<br>Permanent	 10 (55.6)<br>3 (16.7)<br>5 (27.8)	 4 (36.4)<br>2 (18.2)<br>5 (45.5)	 6 (85.7)<br>1 (14.3)<br>0 (0.0)	 0.092

Prior AF ablation (%)	7 (38.9)	4 (36.4)	3 (42.9)	1.000
History of NSVT (%)	16 (14.2)	15 (21.7)	1 (2.3)	0.004
Baseline 5-year risk SCD (%)	1.89 (1.36-2.60)	1.90 (1.40-3.00)	1.78 (1.33-2.40)	0.252
ASA (%)	4 (3.5)	1 (1.5)	3 (6.8)	0.297
Septal myectomy (%)	3 (2.7)	2 (2.9)	1 (2.3)	1.000
Imaging studies				
PLAX LA diameter (mm)	41.5 ± 7.5	42.8 ± 7.6	39.3 ± 7.1	0.017
Maximum LV thickness (mm)	17 (15-20)	17.5 (16-20)	17 (15-20)	0.128
LVEF (%)	66.3 ± 7.0	65.8 ± 7.6	67.0 ± 5.9	0.395
Significant LVOT obstruction (%)	22 (19.5)	12 (17.4)	10 (22.7)	0.485
Apical aneurysm (%)	5 (4.4)	4 (5.8)	1 (2.3)	0.647
Late gadolinium enhancement (%)	58 (67.4)	43 (84.3)	15 (42.9)	<0.001
24-hour Holter monitoring (n=96)				
NSVT (%)	17 (17.7)	16 (25.8)	1 (2.9)	0.005
AF (%)	5 (5.2)	5 (8.1)	0 (0.0)	0.157
Premature atrial complexes (n)	47 (13-220)	87 (15-225)	37 (9-126)	0.349
Premature ventricular complexes (n)	32 (3-181)	37 (9-181)	10 (2-178)	0.195

Abbreviatures: PAD Peripheral artery disease; COPD Chronic obstructive pulmonary disease; ACEi Angiotensin converting enzyme inhibitors; ARB Angiotensin II receptor blockers; P/LP Pathogenic/Likely Pathogenic; HF Heart failure; AF Atrial fibrillation; NSVT Non-sustained ventricular tachycardia; ASA Alcohol septal ablation; LA Left atrium; LV Left ventricle; LVEF Left ventricular ejection fraction; MR mitral regurgitation
Notes: A total of 99 patients (87.6%) underwent genetic testing. Valvular heart disease refers to moderate or severe disease at the mitral or aortic position. Significant LVOT obstruction was defined as ≥ 30 mmHg as per clinical guidelines. A total of 86 patients (76.1%) underwent cardiac magnetic resonance.

Supplementary Table S4 Logistic regression analysis of univariable and multivariable predictors of NSVT

	Univariable	Multivariable

	OR (95% CI)	SE	p value	OR (95% CI)	SE	p value
Age (per 10 years increment)	1.46 (1.11-1.92)	0.20	0.007	1.36 (0.97-1.89)	0.23	0.072
History of NSVT	11.94 (1.52-94.04)	12.58	0.018	8.25 (0.86-79.09)	9.51	0.067
PLAX LA diameter (per mm)	1.07 (1.01-1.13)	0.03	0.021	1.04 (0.97-1.12)	0.04	0.258
LGE in MRI	3.20 (1.45-7.05)	1.29	0.004	2.42 (1.03-5.71)	1.06	0.043
Baseline HCM SCD (per 1%)	1.31 (0.91-1.91)	0.25	0.150	1.04 (0.60-1.79)	0.29	0.890

Abbreviatures: NSVT Non-sustained ventricular tachycardia, PLAX LA Parasternal long axis left atrial, LGE Late gadolinium enhancement, MRI Magnetic resonance imaging.

Supplementary Table S5. Differences between patients with early and non-early NSVT (first 24 hours vs 24h-30 days)

Variable	All patients (n=69)	First 24h (n=10)	24h-30d (n=59)	p value
Male sex (%)	56 (81.2)	8 (80.0)	48 (81.4)	1.000
Age (years)	60.6 (50.8-70.8)	58.5 (53.7-67.0)	60.6 (49.4-71.7)	0.865
Hypertension (%)	34 (49.3)	5 (50.0)	29 (49.2)	1.000
Diabetes (%)	8 (11.6)	1 (10.0)	7 (11.9)	1.000
Dyslipidemia (%)	30 (43.5)	3 (30.0)	27 (45.8)	0.496
Cerebrovascular disease (%)	5 (7.3)	0 (0.0)	5 (8.5)	1.000
PAD (%)	1 (1.5)	0 (0.0)	1 (1.7)	1.000
Chronic kidney disease (%)	2 (2.9)	0 (0.0)	2 (3.4)	1.000
COPD (%)	3 (4.4)	0 (0.0)	3 (5.1)	1.000
Coronary artery disease (%)	5 (7.3)	1 (10.0)	4 (6.8)	0.555
Valvular heart disease (%)	9 (13.0)	2 (20.0)	7 (11.9)	0.609
ACEi/ARB (%)	29 (42.0)	4 (40.0)	25 (42.4)	1.000
Beta blockers (%)	41 (59.4)	7 (70.0)	34 (57.6)	0.729
Dysopiramide (%)	4 (5.8)	0 (0.0)	4 (6.8)	1.000

Other antiarrhythmics (%)	2 (2.9)	1 (10.0)	1 (6.7)	0.271
Antiplatelets (%)	9 (13.0)	2 (20.0)	7 (11.9)	0.609
Anticoagulation (%)	12 (17.4)	3 (30.0)	9 (15.3)	0.362
Family history of SCD (%)	10 (14.5)	0 (0.0)	10 (17.0)	0.337
P/LP variant carrier (%)	26 (41.3)	5 (55.6)	21 (38.9)	0.469
Proband status (%)	53 (76.8)	8 (80.0)	45 (76.3)	1.000
NYHA class (%)<br>I<br>II<br>III	 48 (69.6)<br>19 (27.5)<br>2 (2.9)	 8 (80.0)<br>2 (20.0)<br>0 (0.0)	 40 (67.8)<br>17 (28.8)<br>2 (3.4)	0.790
Prior HF decompensations (%)	6 (8.7)	0 (0.0)	6 (10.2)	0.582
Prior syncope (%)	4 (5.8)	0 (0.0)	4 (6.8)	1.000
Palpitations (%)	18 (26.1)	1 (10.0)	17 (28.8)	0.274
Prior atrial fibrillation (%)	11 (15.9)	3 (30.0)	8 (13.6)	0.192
Type of AF (%)<br>Paroxysmal<br>Persistent<br>Permanent	 4 (36.4)<br>2 (18.2)<br>5 (45.4)	 0 (0.0)<br>0 (0.0)<br>3 (100.0)	 4 (50.0)<br>2 (25.0)<br>2 (25.0)	0.139
Prior AF ablation (%)	4 (36.4)	0 (0.0)	4 (50.0)	0.236
History of NSVT (%)	15 (21.7)	6 (60.0)	9 (15.3)	0.005
Baseline 5-year risk SCD (%)	1.90 (1.40-1.30)	3.41 (1.83-4.05)	1.78 (1.27-2.71)	0.030
ASA (%)	1 (1.5)	0 (0.0)	1 (1.7)	1.000
Septal myectomy (%)	2 (2.9)	1 (10.0)	1 (1.7)	0.271
Imaging studies				
PLAX LA diameter (mm)	42.8 ± 7.6	46.1 ± 8.6	42.2 ± 7.3	0.138
Maximum LV thickness (mm)	17 (15-20)	17 (17-21)	17 (15-20)	0.365
LVEF (%)	65.8 ± 7.6	65.2 ± 6.4	65.9 ± 7.8	0.781
Significant LVOT obstruction (%)	12 (17.4)	1 (10.0)	11 (18.6)	0.679
Apical aneurysm (%)	4 (5.8)	1 (10.0)	3 (5.1)	0.474

Late gadolinium enhancement (%)	43 (84.3)	9 (100.0)	34 (81.0)	0.322
24-hour Holter monitoring (n=62)				
NSVT (%)	16 (25.8)	7 (70.0)	9 (17.3)	0.002
AF (%)	5 (8.1)	3 (30.0)	2 (3.9)	0.026
Premature atrial complexes (n)	87 (15-225)	19 (13-82)	146 (20-230)	0.071
Premature ventricular complexes (n)	37 (9-181)	163 (38-895)	29 (7-172)	0.063

Abbreviatures: PAD Peripheral artery disease; COPD Chronic obstructive pulmonary disease; ACEi Angiotensin converting enzyme inhibitors; ARB Angiotensin II receptor blockers; P/LP Pathogenic/Likely Pathogenic; HF Heart failure; AF Atrial fibrillation; NSVT Non-sustained ventricular tachycardia; ASA Alcohol septal ablation; LA Left atrium; LV Left ventricle; LVEF Left ventricular ejection fraction; MR mitral regurgitation
Notes: A total of 99 patients (87.6%) underwent genetic testing. Valvular heart disease refers to moderate or severe disease at the mitral or aortic position. Significant LVOT obstruction was defined as ≥ 30 mmHg as per clinical guidelines. A total of 86 patients (76.1%) underwent cardiac magnetic resonance.

Supplementary Figure S1

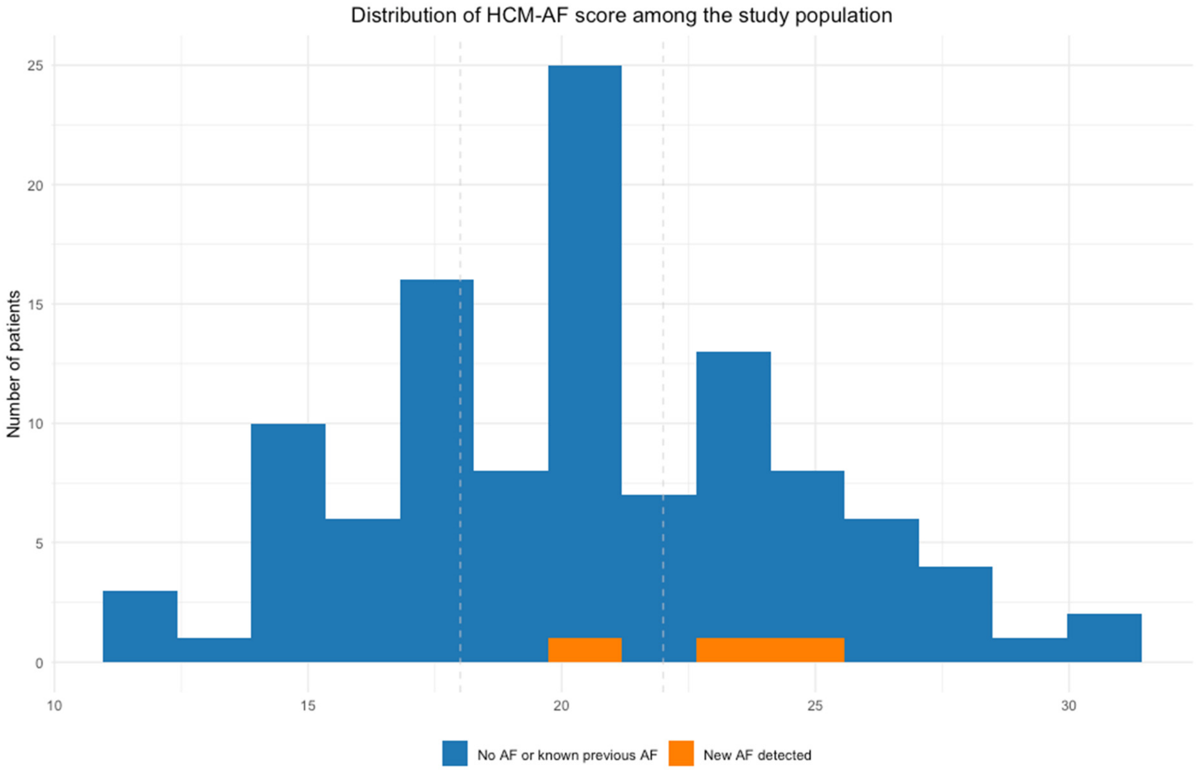

Supplement: Supplementary file 1 [file jcm-14-07432-s001.zip › jcm-3909781-supplementary.pdf]
